# An fMRI Study of the Brain Network Involved in Teeth Tapping in Elderly Adults

**DOI:** 10.3389/fnagi.2020.00032

**Published:** 2020-03-17

**Authors:** T. Kobayashi, H. Fukami, E. Ishikawa, K. Shibata, M. Kubota, H. Kondo, Y. Sahara

**Affiliations:** ^1^Department of Prosthodontics and Oral Implantology, School of Dentistry, Iwate Medical University, Morioka, Japan; ^2^Department of Physiology, School of Dentistry, Iwate Medical University, Shiwa-gun, Japan; ^3^Department of Oral Health Sciences, Faculty of Nursing and Health Care, Baika Women’s University, Osaka, Japan

**Keywords:** fMRI, teeth tapping, denture, sensory input, voluntary movement

## Abstract

Cortical activity during jaw movement has been analyzed using various non-invasive brain imaging methods, but the contribution of orofacial sensory input to voluntary jaw movements remains unclear. In this study, we used functional magnetic resonance imaging (fMRI) to observe brain activities during a simple teeth tapping task in adult dentulous (AD), older dentulous (OD), and older edentulous subjects who wore dentures (OEd) or did not wear dentures (OE) to analyze their functional network connections. (1) To assess the effect of age on natural activation patterns during teeth tapping, a comparison of groups with natural dentition—AD and OD—was undertaken. A general linear model analysis indicated that the major activated site in the AD group was the primary sensory cortex (SI) and motor cortex (MI) (*p* < 0.05, family wise error corrected). In the OD group, teeth tapping induced brain activity at various foci (*p* < 0.05, family wise error corrected), including the SI, MI, insula cortex, supplementary motor cortex (SMC)/premotor cortex (PMA), cerebellum, thalamus, and basal ganglia in each group. (2) Group comparisons between the OD and OEd subjects showed decreased activity in the SI, MI, Brodmann’s area 6 (BA6), thalamus (ventral posteromedial nucleus, VPM), basal ganglia, and insular cortex (*p* ¡ 0.005, uncorrected). This suggested that the decreased S1/M1 activity in the OEd group was related to missing teeth, which led to reduced periodontal afferents. (3) A conjunction analysis in the OD and OEd/OE groups revealed that commonly activated areas were the MI, SI, cerebellum, BA6, thalamus (VPM), and basal ganglia (putamen; *p* < 0.05, FWE corrected). These areas have been associated with voluntary movements. (4) Psychophysiological interaction analysis (OEd vs OE) showed that subcortical and cortical structures, such as the MI, SI, DLPFC, SMC/PMA, insula cortex, basal ganglia, and cerebellum, likely function as hubs and form an integrated network that participates in the control of teeth tapping. These results suggest that oral sensory inputs are involved in the control of teeth tapping through feedforward control of intended movements, as well as feedback control of ongoing movements.

## Introduction

Orofacial muscles are involved in several behaviors, including elemental (e.g., tongue protrusion and jaw opening) and semiautomatic (e.g., mastication and swallowing) movements. Many tasks demand complex refined control of the musculature. Animal studies have indicated that chewing involves interactions between sensory feedback and an intrinsic rhythmical neural pattern. Signals from cortical masticatory areas trigger the central pattern generator and execute a coordinated rhythmical jaw movements (for reviews, see Dubner et al., 1978; Lund and Kolta, 2006; Avivi-Arber et al., 2011; Morquette et al., 2012; Avivi-Arber and Sessle, 2018). Although subcortical and cortical motor regions play central roles in orofacial movements, the underlying brain circuitry that initiates and brings about jaw movements remains poorly understood.

Direct investigation of the human cerebral cortical areas involved in jaw movements is possible due to the advent of non-invasive visualizing techniques such as fMRI, PET, and MEG. Initial PET-based exploration of the cortical areas related to mastication showed that the sensorimotor areas of the cortex were strongly activated and this was followed by activation of the supplementary motor areas, insulae, striatum, and Cb (Momose et al., 1997). Using fMRI, patterns of brain activation associated with mandibular movements (Ishikawa, 2002) during teeth clenching (Tamura et al., 2002; Byrd et al., 2009; Iida et al., 2010) and chewing (Onozuka et al., 2002; Tamura et al., 2003) have been identified, and there is agreement that an experimental task evoked concurrent activity in multiple brain regions. Studies that relate a behavior to activity in a single area or to a specific connection have been faced with difficulties. Neither the interconnections of a network have been identified, nor is it known whether these areas comprise a cohesive functional network.

In elderly adults, reduced masticatory performance is thought to be associated with changes in brain structures and/or functions. The MRI methods have been providing important evidences on how the brain changes with age at various gross anatomical and functional levels (Grady, 2012): (1) Healthy aging is known to be associated with GMV reductions and functional alterations in several regions that are crucial for higher cognitive function, i.e., the prefrontal, medial temporal, and parietal cortices. (2) Diffusion MRI methods have shown age-related changes in white matter connectivity between prefrontal and posterior cortical regions and within posterior sensory cortices. Therefore, reduced activity in elderly adults can be assumed to reflect a reduced level of functioning (Grady, 2012). On the other hand, functional MRI studies have also shown that there are additional cortical activations (for reviews, see D’Esposito et al., 2003; Humbert et al., 2009; Grady, 2012). Because synchronized and coherent fluctuations of BOLD signals have been shown to be important for studying underlying neuronal networks of the human brain (Fox and Raichle, 2007), some recent investigations have begun to assess the brain networks and identify connections between these areas (Quintero et al., 2013; Lin et al., 2017; Michely et al., 2018).

The aim of this study was to understand the neural control mechanism of a complex behavior such as orofacial movement. While older human subjects performed a simple dental tapping task in an MRI environment, we examined how subcortical and cortical areas are activated and how they function together to produce behavior. In this study, we applied the PPI analysis for assessing the functional relevance of tapping task-activated areas. We predicted that the effect of denture wearing (peripheral sensory inputs) influences voluntary motor responses in the cortex during the teeth tapping task. fMRI allowed confirmation of the basic findings that have been characterized in non-human animal studies, and these findings have been extended to behaviors observed in humans.

## Materials and Methods

### Subjects

Three groups of healthy subjects were included in this study: (1) an AD group (5 males and 4 females, mean age = 30.7 years, range: 26–40 years); (2) an OD group (6 males and 7 females, mean age = 81.8 years, range: 80–83 years); and (3) an OEd group (7 males and 8 females, mean age = 79.1, range: 71–88 years). To reduce unidentified variance between the subject groups, careful characterization and selection of the subjects was undertaken. All subjects (1) were right-handed; (2) cleared the International Classification of Impairments, Disabilities, and Handicaps test and the Active-Daily Living Score (ADI Score) test; (3) had no history of neurologic or psychiatric disease; and (4) had no history of oral surgery, dental implants, head and neck cancer, and temporomandibular joint disorders. The dentitional states of the subjects in the OD group were Eichner A-1 type. The subjects’ dentures were fabricated using traditional techniques, and all patients were satisfied with their daily denture use at the point of examinations (∼3 months). After a full de-briefing, written informed consent was obtained from each subject. The experimental protocol for the use of human subjects was approved by the Medical Ethics Committee of Iwate Medical University (approval # 01071 and 01254).

### Task Paradigm

**FIGURE 1:**
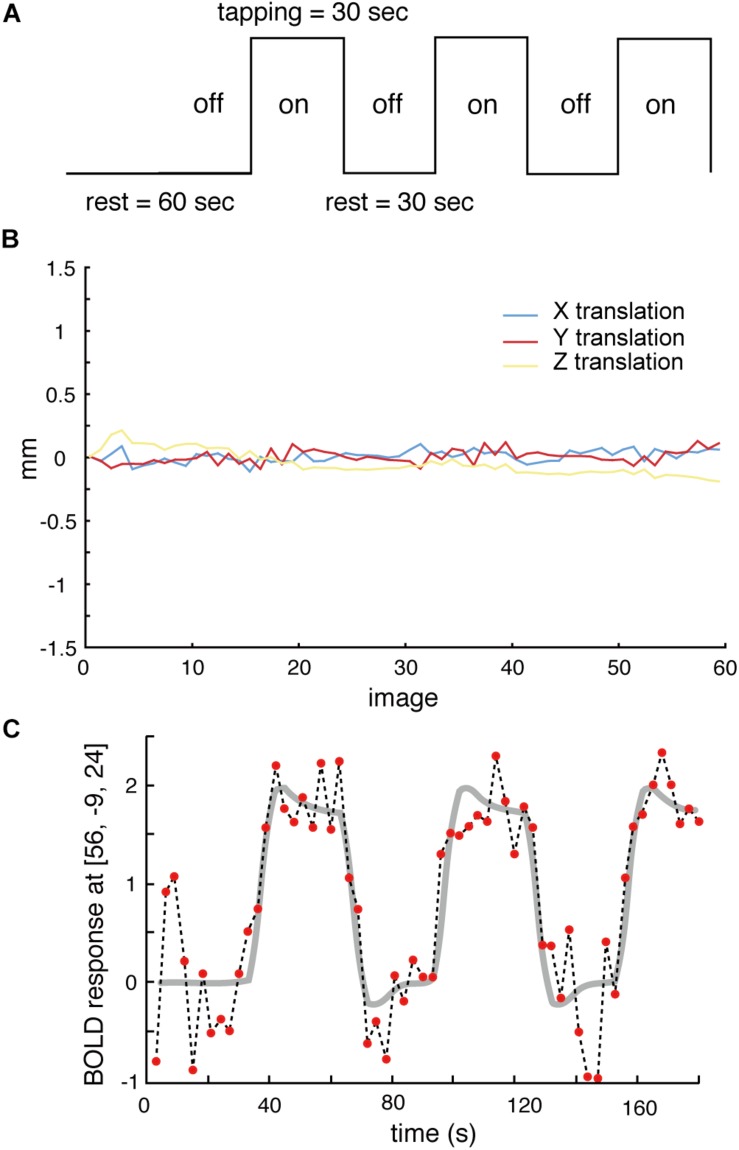
**(A)** The task paradigm is represented by periods of teeth tapping. During the measurements, subjects alternated between 30 s of rest (off) and 30 s of tapping (on). The on–off cycle was repeated three times during each scanning run. **(B)** All data from subjects whose heads were evaluated to have moved >1 mm, as determined by a free software package (SPM8) that calculated head motion, were discarded. **(C)** Example BOLD signals sampled at the MI [56, −9, 24] from one participant. Changes in signal intensity with time on an image-by-image basis 60 successive images during three cycles of tapping. The vertical axis shows the BOLD signal intensity in arbitrary units.

During scanning, participants lay on their backs in the scanner. They were instructed (1) to keep the lower jaw in a rest position during the rest stage and (2) to perform a teeth tapping task, i.e., light tapping of the upper and lower teeth together with self-paced timing (∼1 Hz). We employed a block design in this experiment (Figure 1A). The task comprised 30 s in a resting position (off) followed by 30 s of teeth tapping (on), i.e., continuous tapping throughout the entire 30 s block. This task was repeated three times in a run. Therefore, a total duration of each scan was 210 s (dummy 30 s + task 180 s). A single fMRI trial included two runs for the AD and OD groups. The OE group performed the task with and without dentures. Within these trials, the order was randomized yet equal for both conditions to counter ordering effects and to maintain comparability. During the tapping task without dentures (sham teeth tapping), the subjects were instructed to mimic the teeth tapping movements, but were told not to bring the gums into contact. For data analysis, the first 10 volumes were discarded because of unstable magnetization. To minimize motions during the measurements, the head of the examinee was fixed in a supine position. All data from subjects whose head moved >1 mm, which was determined by a motion correction procedure in SPM8 (Figure 1B), were rejected. Imaging data of four elderly subjects (two males and two females of OE/OEd group) were excluded from all analyses.

### Image Acquisition

Adults and two groups of elderly adult subjects (aged > 80 years); i.e., people with >20 teeth remaining (OD group) and the OE/OEd group, underwent fMRI during the teeth tapping task using EPI with a 3T MRI scanner. For each subject, functional images were acquired with a 3.0-T Signa HDxt system (GE Medical Systems, Milwaukee, WI, United States). The fMRI data were acquired using the gradient-EPI sequence (Bandettini et al., 1992) with the following BOLD imaging parameters: each volume comprised 24 oblique slices (in the axial orientation), each slice was 5 mm thick, and there was no gap. The TR was 3000 ms with a FA of 60° and 30 ms of TE. The FOV was 240 mm and the in-plane matrix size was 64 × 64 pixels (i.e., resolution 3.75 × 3.75 × 5 mm). T1-weighted images for anatomical details were obtained with the 3D-SPGR pulse sequence: TR 7.584 ms; TE 1.56 ms; FOV 240 mm; matrix 128 × 128; and 148 slices. The resolution of T1-weighted images was 1.875 × 1.875 × 1.4 mm.

### Data Analysis

The MRI data were converted to an ANALYZE format and examined using MATLAB (R2007b, Mathworks, Natick, MA, United States) and SPM8 (Wellcome Department of Cognitive Neurology, London, United Kingdom, http://www.fil.ion.ucl.ac.uk/spm/).

First, 60 images from each trial were automatically realigned to the first image to correct for movements (Friston et al., 1994, 1995, 1998). Then, the images from two runs were co-registered with the T1-weighted anatomical image and normalized into a standard stereotaxic space that corresponded to the MNI template (provided by the MNI; Evans et al., 1994). To increase the signal-to-noise ratio, all functional images were smoothed with an 8-mm full-width at half-maximum isotropic Gaussian kernel filter.

### General Linear Model

Statistical analyses were based on the GLM (Friston et al., 1995). Data were analyzed for each subject individually in the first level of analysis and individual contrast images were used for a random-effects analysis that assessed group effects. The statistical threshold for comparisons of the main effect of each condition was set (*p* < 0.05) using the FWE correction provided by SPM8. The cluster level was set at 10.

The resulting areas of activation were characterized regarding peak height and spatial extent (>10 voxels). Functional images were generated based on a two-tailed *t-*test that compared the resting and active conditions on a pixel-by-pixel basis. The results were considered significant at *p* < 0.05. The FWE rate corrected for the whole brain volume. Significant voxels were expressed with their coordinates in Talairach’s space transformed from MNI coordinates (Talairach Daemon client software version 1.1; Research Imaging Center, University of Texas Health Science Center). Anatomical maps were created using the Anatomy Toolbox to identify the location, intensity, and activated voxels in the anatomical regions. Images were first evaluated at the single-subject level and then group statistics were computed. The activation regions of the three groups were determined and compared using a one-way ANOVA. Conjunction analysis was performed to analyze the brain regions activated in all three groups.

### PPI Analysis

To test the increase in sensory input induced by denture wearing on functional connectivity, PPI analyses were performed. PPI analysis provides a way to determine how neural activity from a seed region interacts with a psychological state to stimulate different levels of neural activity in other parts of the brain (Friston et al., 1997; Bingel et al., 2007; Kim and Horwitz, 2008). Subject-specific seed regions were determined by analyzing each OE individual subject’s contrasts for tapping with dentures (OEd) and no dentures (OE, sham tapping) displayed at *p* < 0.05 referring for a value of conjunction analysis. Seed regions were defined as 10-mm spheres around the peak voxel rate of the activated cluster. The psychological variable was defined as the contrast of denture wearing vs no-denture wearing. In the first-level analysis, the PPI regressor was extracted from each subject. The PPI regressor consisted of the convolution of two functions, i.e., hemodynamic function convolved task regressor (denture wearing vs no-denture wearing) and the BOLD time course extracted from the seed ROI. This regressor was used to identify the individual effect of sensory modulation on connectivity. The second-level analysis was a group-level analysis. PPI results are reported at a statistical threshold of *p* < 0.005 (uncorrected) with a cluster size threshold of five voxels.

## Results

### Comparison of Teeth Tapping in Young and Elderly Adults

**FIGURE 2:**
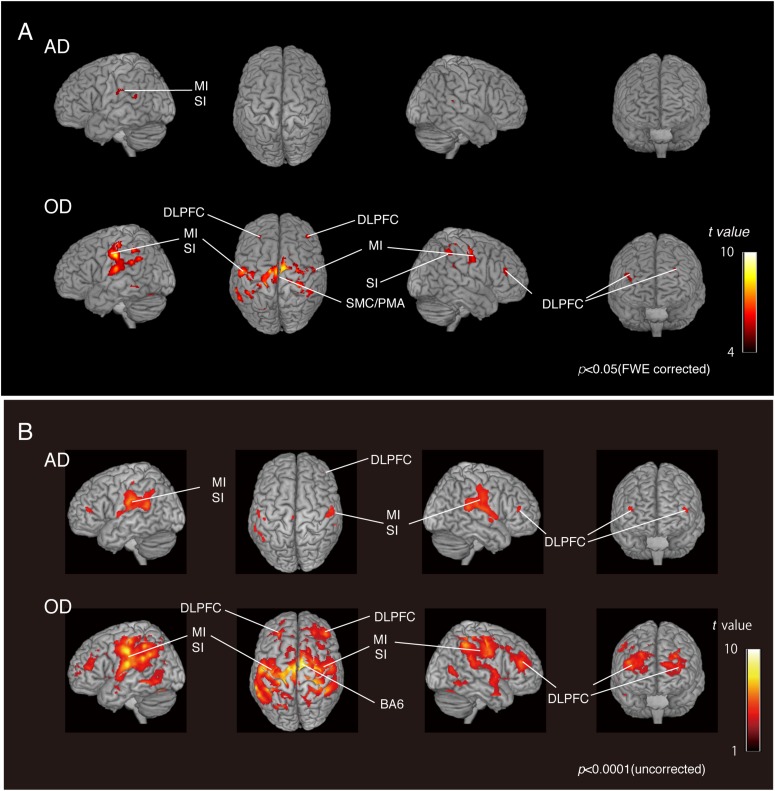
BOLD activation pattern. **(A)** Surface projection of statistical parametric maps superimposed onto a standard MNI template brain (*p* < 0.05, FWE corrected). Significant increases in signals associated with tapping in the AD group **(upper section)** and OD group **(lower section)**. The color bar shows the *t*-values. **(B)** Surface projection of statistical parametric maps superimposed onto a standard MNI template brain (*p* < 0.0001, uncorrected). Significant increases in signals associated with tapping in the AD group **(upper section)** and OD group **(lower section)**. The color bar shows the *t*-values. Activated regions from uncorrected results provide some additional cortical activations to FWE corrected results **(A)**, suggesting that the vast number of statistical tests may lead to false positive results regardless of the analysis approach used.

We compared the two groups with natural dentition—AD and OD—to assess the effect of age on natural activation patterns during teeth tapping. As shown in Figure 2A (upper), the major activated site in the AD group was the primary sensory cortex and motor cortex. Small sites of activation were found in the association cortex. In the OD group, additional areas were activated and then compared with the AD group [Figure 2A (lower)]. The activated areas that were found bilaterally were the motor/somatosensory cortices, temporal association cortex, and parietal association cortex.

As shown in the Figure 2B, activated regions from uncorrected results provided some additional cortical activations to FWE-corrected results, as has been seen in other fMRI studies (Onozuka et al., 2003; Fang et al., 2005). Since fMRI studies rely on the detection of a weak signal in the presence of substantial noise, many statistical tests may lead to false-positive results regardless of the analytical approach. However, it has also been pointed out that standard corrections, such as Bonferroni’s methods, are too strict and may eliminate true activation.

### MRI Results During a Teeth Tapping Task in the OD and OE Groups

**FIGURE 3:**
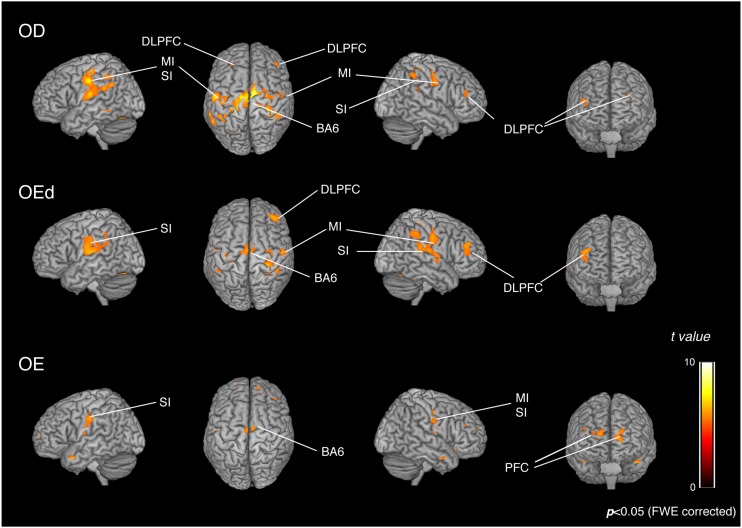
Teeth tapping induced activated regions on the cortex in the OD **(top)**, OEd **(middle)**, and OE **(bottom)** groups. Surface projection of statistical parametric maps (GLM analysis) superimposed onto a standard MNI template brain (*p* < 0.05, FWE corrected). The color bar shows the *t*-values. Teeth tapping activated various foci, including the sensorimotor cortex, BA6, and prefrontal cortex.

**FIGURE 4:**
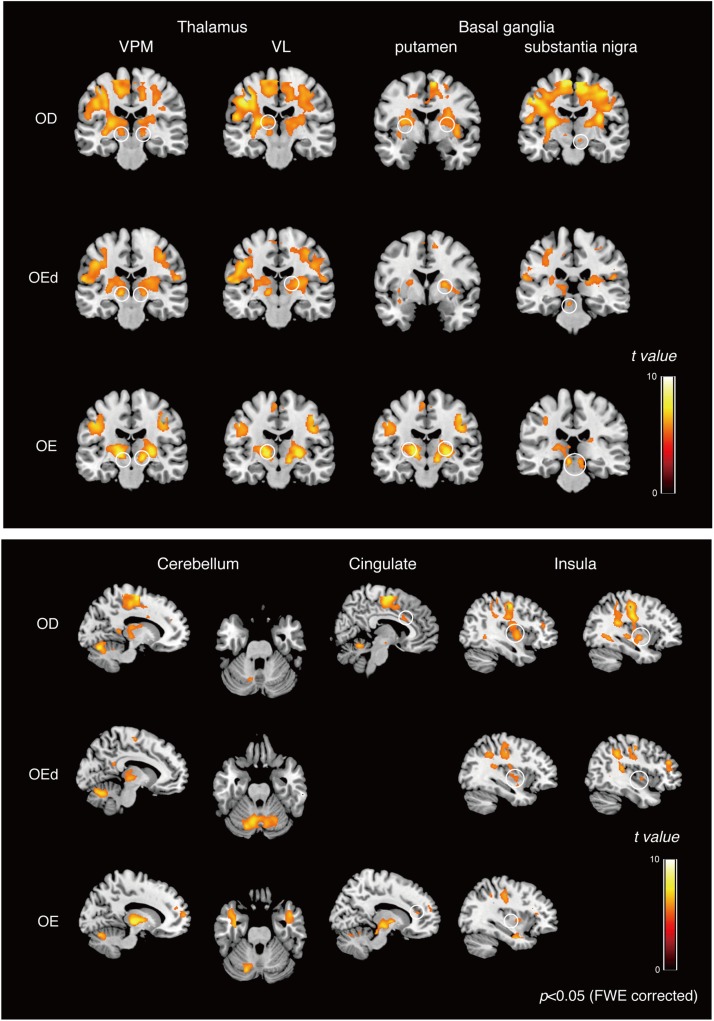
Teeth tapping activated foci in the cortical and subcortical areas in the OD **(top)**, OEd **(middle)**, and OE **(bottom)** groups. Results from GLM analysis were superimposed onto a standard MNI template brain (*p* < 0.05, FWE corrected). The color bar shows the *t*-values. Activated regions, the thalamus (VPM and VL), basal ganglia (putamen and substantia nigra, shown on the coronal planes), cerebellum (shown on the sagittal and coronal planes), cingulate, and insula (shown on the sagittal planes). The color bar shows the *t*-values.

Statistical maps of brain regions with significant increases in BOLD contrast during a teeth tapping task in the OD and OE groups are shown in Figures 3, 4, respectively. Teeth tapping tasks in elderly adults recruited various subcortical and cortical foci, and the locations of the most significant foci of activation are summarized in Table 1, in which MNI coordinates of anatomical regions with maximum *t-*values are shown. Activated areas of the OD group were found bilaterally in the SI, MI, prefrontal cortex, temporal association area, and SMC. Activation foci were also revealed in cortical areas such as the insula (INS) and cingulate gyrus. Significant subcortical activation was present in the putamen and substantia nigra of the Bg, ventral posteromedial nucleus (VPM), and ventral lateral (VL) nucleus of the Th, amygdala, and cerebellar cortex. However, tapping tasks in the OE group recruited relatively smaller areas in the MI, SI, BA6, and prefrontal cortex than the OD group. The dorso-lateral area of the prefrontal cortex (DLPFC) was activated in the OD and OEd groups, but not in the OE groups.

**TABLE 1:** Significantly activated regions during tapping in the AD, OD, OE, and OEd groups.

	Activated area	Brodmann’s area	MNI coordinates			*t*-value
			*x*	*y*	*z*	
AD	Primary motor cortex (MI)	4	−51	−19	24	4.77
			47	−12	25	4.69
	Primary sensory cortex (SI)	1, 2, 3	−47	−45	16	5.62
			−45	−25	30	6.51
OD	Primary motor cortex (MI)	4	−47	−16	37	6.76
			32	−36	54	7.3
	Primary sensory cortex (SI)	1, 2, 3	−39	−16	52	7.07
			33	−39	54	7.34
	Supplementary/premotor area	6	−11	−12	61	6.71
			8	−7	61	6.48
	Thalamus					
	VPM		−14	−22	−2	6.03
			15	−23	−3	5.66
	VL		−14	−19	9	5.41
	Basal ganglia					
	Putamen		−29	−2	9	5.97
			21	−2	12	5.73
	Substantia nigra		8	−13	−11	4.87
	DLPFC	9	−27	29	30	4.99
	Cerebellum		−14	−65	−28	5.73
	Cingulate cortex	24	6	16	27	5.59
	Insula		38	−3	0	6.48
			−36	−5	−4	6.15
OE	Primary motor cortex (MI)	4	−47	−16	37	5.78
			45	−10	28	5.76
	Primary sensory cortex (SI)		28	−21	42	5.26
		1, 2, 3	39	−18	42	6.43
	Supplementary/premotor area	6	−5	−10	60	5.19
			8	−7	63	5.25
	Thalamus					
	VPM		−15	−16	0	6.49
			15	−18	−3	6.00
	VL		−15	−14	2	8.23
	Basal ganglia					
	Putamen		26	−15	1	6.18
	Substantia nigra		−6	−25	−9	7.10
	Cerebellum		−14	−65	−26	
	Cingulate cortex	24	12	37	15	5.44
	Insula		−35	0	1	5.61
OEd	Primary motor cortex (MI)	4	−51	−12	24	6.96
			53	−12	39	6.70
	Primary sensory cortex (SI)	1, 2, 3	−45	−18	36	7.02
			32	−36	52	6.72
	Supplementary/premotor area	6	45	−12	30	7.07
			−3	−10	−61	6.56
	Thalamus					
	VPM		−13	−19	1	6.57
			15	−21	−1	6.39
	VL		15	−17	10	5.47
	Basal ganglia					
	Putamen		22	0	7	6.31
			−22	3	7	5.22
	Substantia nigra		−4	−27	−15	6.17
	DLPFC	9	41	41	24	6.70
	Cerebellum		−10	−63	−24	7.13
	Insula		40	−3	−2	5.57
			−36	−3	−2	5.53

*p < 0.05 FWE corrected.*

### Differences in Teeth Tapping in the OD and OEd Groups

**FIGURE 5:**
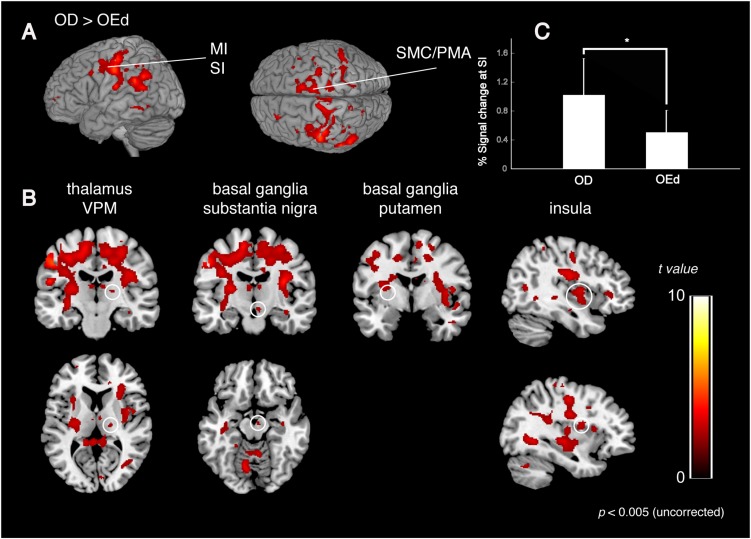
Comparison between OD and OEd groups. **(A,B)** Results from group comparisons between OD and OEd (OD > OEd) are shown. Activated areas were decreased in the MI, SI, thalamus (VPM), basal ganglia, and insula cortex (*p* < 0.005, uncorrected). The color bar shows the *t*-values. **(C)** The reduction of BOLD response strengths in the SI (*p < 0.05). The reduction of representation areas and BOLD response strengths can be related to missing teeth, which leads to reduced periodontal afferents.

We compared the brain activation patterns in the OEd to OD groups while they were performing the teeth tapping tasks. Group comparison analysis (OD > OEd) showed that activated areas were different in the SI, MI, SMC, Th (VPM), Bg (putamen and substantia nigra), and insula (Figures 5A,B; for coordinates and *t*-values, see Table 2).

**TABLE 2:** Significantly activated regions during tapping (OD > OEd).

	Activated area	Brodmann’s area	MNI coordinates			*t*-value
			*x*	*y*	*z*	
OD - OEd	Primary motor cortex (MI)	4	−51	−15	41	4.98
	Primary sensory cortex (SI)	1, 2, 3	−45	−16	51	4.42
	Supplementary/premotor area	6	12	−14	62	2.96
	Thalamus					
	VPM		−18	−17	8	2.36
	Basal ganglia					
	Putamen		−29	−2	9	2.24
	Substantia nigra		5	−12	−14	2.56
	Insula		39	−2	6	3.12
			−35	0	15	2.67

*p ¡ 0.005 uncorrected.*

The initial representation of oral somatosensory input is strictly somatotopically organized with the orofacial region (face SI) represented most laterally (Haggard and de Boer, 2014). In accord with the functional representation in the precentral gyrus, where the superior–inferior gradient was established by the sensory homunculus with the lips located dorsally to the teeth and the teeth dorsally to the tongue, the areas representing maximum tapping without dentures tended to be observed inferio-anterior to those while tapping with dentures. In addition, the mean percent signal changes also demonstrated significant between-group differences in the SI regions (Figure 5C). Thus, the reduction in brain map size and reduced response strength are best explained as that caused by reduced sensory inputs (Godde et al., 2002) and could be related to missing teeth, which leads to reduced periodontal afferents.

### Identification of Common Activated Areas in the OD and OEd/OE Groups

**FIGURE 6:**
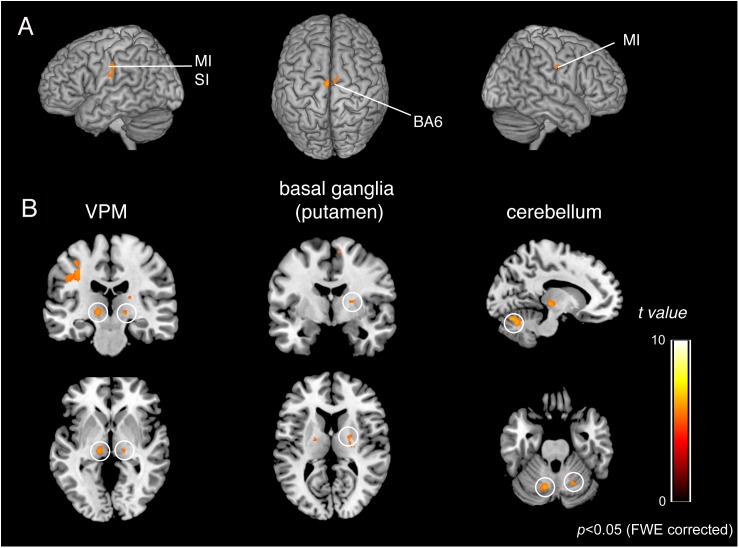
BOLD responses common to teeth tapping tasks among OD, OE, and OEd, as determined by a group conjunction analysis (*p* < 0.05, FWE corrected). Regions of significant activation are shown on cortical surface projections **(A)** and normalized coronal, axial, and sagittal brain slices **(B)**. Areas include the sensorimotor cortex, BA6, cerebellum, thalamus (VPM and VL), and basal ganglia (putamen). The color bar shows the *t*-values.

To identify the brain regions that were activated during tapping tasks in the OD and OEd/OE groups, a conjunction analysis (Friston et al., 1999) was conducted. A conjunction analysis revealed that teeth tapping induced activation in the MI, SI, SMC/PMA, VPM in the Th, Cb, and putamen in the Bg (*p* < 0.05, FWE corrected), as shown in Figure 6 and Table 3 (for coordinates and *t*-values). These areas have been shown to be regions that are involved in voluntary movements of primates. MI and PMA/SMC have been suggested to be involved in motor planning and initiation (for reviews, see Hoshi and Tanji, 2007; Nachev et al., 2008), and the Bg and Cb are well-known subcortical centers that provide a loop that is essential to the smooth execution of skilled movements (Caligiore et al., 2017; Bostan and Strick, 2018). Furthermore, preparatory activity has been identified in the MI, premotor and supplemental motor cortex, parietal cortex, frontal eye field, striatum, motor-related Th, superior colliculus, and Cb in non-human primates (Svoboda and Li, 2018).

**TABLE 3:** Significantly activated regions during tapping (conjunction analysis).

	Activated area	Brodmann’s area	MNI coordinates			*t*-value
			*x*	*y*	*z*	
Conjunction	Primary motor cortex (MI)	4	−51	−10	23	6.26
			45	−12	30	6.18
	Primary sensory cortex (SI)	1, 2, 3	−51	−15	24	6.26
	Supplementary/premotor area	6	9	−5	63	5.77
	Thalamus					
	VPM		−14	−21	−2	6.1
			15	−8	−2	5.63
	Basal ganglia					
	Putamen		−20	−5	11	5.39
			20	−5	−26	5.61
	Cerebellum		−14	−66	−27	6.44
			21	−62	−27	5.92
			−51	−10	23	6.26

*p < 0.05 FWE corrected.*

### Functional Connectivity Analysis During the Dental Tapping Task

**FIGURE 7:**
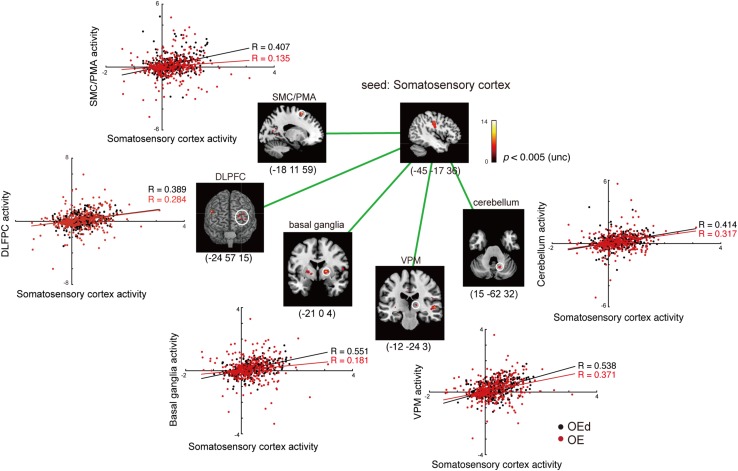
Results of the functional connectivity/PPI analysis. Tapping with denture wearing enhanced the effective connectivity from the SI to the thalamus (VPM), basal ganglia (putamen), SMC/PMA (=BA6), cerebellum, and DLPFC (*p* < 0.05, uncorrected). The location superimposed on a template of the seed region and the target regions shows a response that was significantly associated with stronger connectivity with the seed region under OE (red dots) vs OEd (black dots) conditions. The color bar shows *t*-values. Scatterplots show the regression of activity in the VPM, basal ganglia (putamen), BA6, cerebellum, and DLPFC on the activity in the seed region SI/MI during tapping in the OE vs OEd groups. For each condition, the correlation coefficient between the activity in the seed and target is indicated.

**FIGURE 8:**
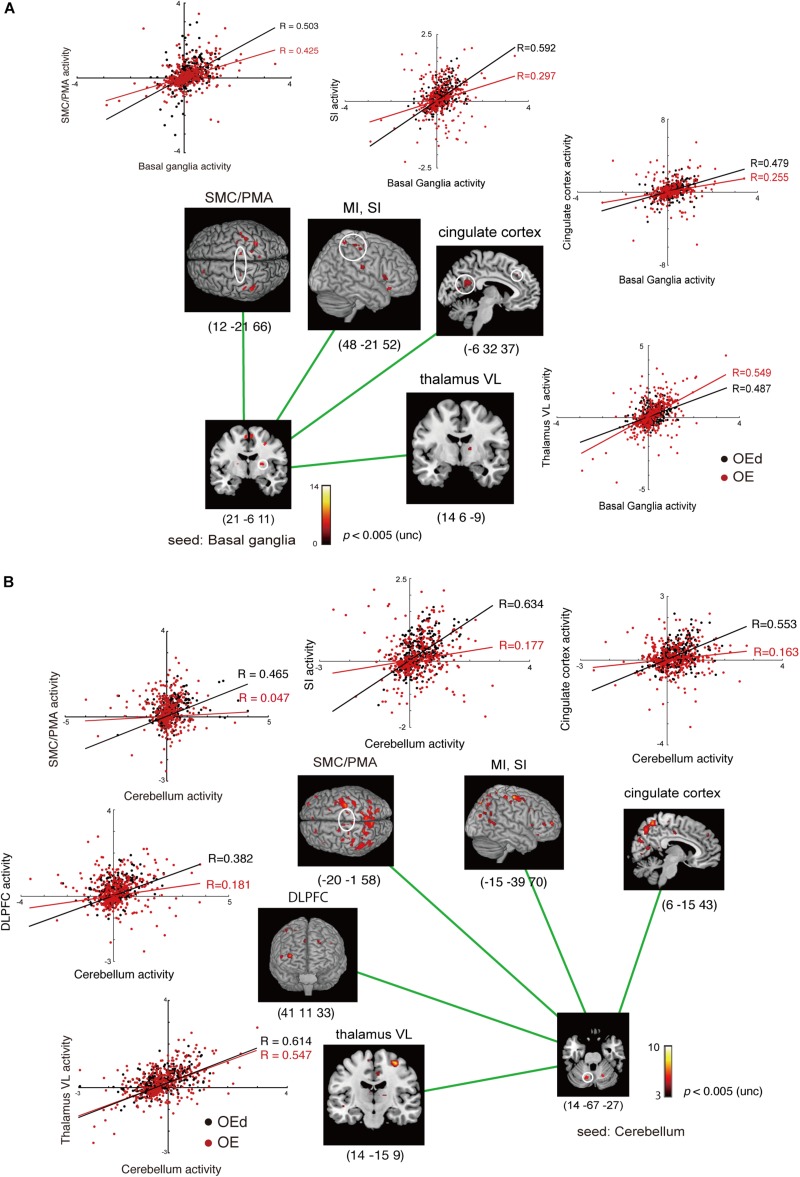
Results of the functional connectivity/PPI analysis. The location superimposed on a template of the seed regions [basal ganglia **(A)** and cerebellum **(B)**] and the target regions shows neural responses that were significantly associated with stronger connectivity with the seed region under OE vs OEd conditions. For each condition, the correlation coefficient between the activity in the seed and targets is indicated. The color bar shows *t*-values. Co-activation plot in panel **(A)** shows the changes in coupling between dentures in vs dentures out; activity in the SI/MI, SMC/PMA, thalamus (VL), and cingulate cortex becomes more sensitive to inputs from the basal ganglia (putamen) with dentures. Co-activation plot in panel **(B)** shows that the activity in the cerebellum positively predicted activity in the VL, DLPFC, SMC/PMA (=BA6), SI/MI, and cingulate cortex with dentures.

To determine the functional relevance of tapping task-activated areas, we performed a PPI analysis. The effect of denture wearing on connectivity modulations was assessed; i.e., our prediction was that peripheral sensory inputs influence voluntary motor responses in the cortex during tapping tasks. Subject-specific seed regions were identified by searching for tapping contrasts for each OE individual subject with dentures in (OEd) and dentures out (OE, sham tapping), evaluated at *p* < 0.05 referring for a value of conjunction analysis. Seed regions included the MI, SI, PMA/SMC, putamen in the Bg, and cerebellar cortex (Figures 7, 8 and Table 4).

**TABLE 4:** Anatomical location and MNI coordinates of PPI analysis (OEd vs OE; *p* < 0.005 uncorrected on the cluster level).

		MNI coordinates			
	Activated area	*x*	*y*	*z*	*t*-value
PPI	Supplementary/premotor area (BA6)	−18	11	59	10.05
seed: left SI	Thalamus				
	VPM	−12	−24	3	5.05
		14	−23	6	4.02
	Basal ganglia				
	Putamen	18	−2	5	12.55
		−21	0	4	4.22
	Cerebellum	15	−62	32	6.25
	DLPFC	−24	57	15	5.67
PPI	Supplementary/premotor area (BA6)	12	−21	66	4.63
seed: right BG	Thalamus				
	VL	14	−6	−9	4.4
	MI	38	−30	59	4.18
	SI	48	−21	52	4.25
	Cingulate cortex	−6	32	37	3.77
		51	−18	22	5.77
PPI	Supplementary/premotor area (BA6)	32	−15	60	8.12
seed:	Thalamus				
left cerebellum	VL	14	−15	9	3.58
	MI	−3	−19	67	3.58
	SI	−15	−39	70	3.58
	Cingulate cortex	6	−15	43	3.88
	DLPFC	41	11	33	4.25
		−20	42	37	4.42
PPI	Cerebellum	−11	−75	−17	3.78
seed:	VPM	15	−24	2	3.55
right BA6					
PPI	SI	47	−25	56	3.45
seed:	Supplementary/premotor area (BA6)	8	−20	63	5.34
left VPM					
PPI	Supplementary/premotor area (BA6)	−36	8	54	4.84
seed:	SI	−41	−25	51	4.58
left insula	Cingulate cortex	−2	−1	36	4
	PFC	−42	41	16	3.66

*p < 0.005 uncorrected*

First, we seeded the SI region for PPI analysis. The responses of the Th (VPM), Bg (putamen), and PMA/SMC showed a stronger connectivity with the SI under OE conditions versus OEd conditions. As shown in Figure 7, tapping with dentures enhanced the connectivity to the VPM nuclei in the Th, putamen in the Bg, SMC, Cb, and the DLPFC (*p* < 0.05). The correlation coefficient between the activity in a seed (SI) and targets was shown under OE (red dots) conditions versus OEd (black dots) conditions.

By seeding the VPM nucleus of the Th for PPI analysis, we confirmed that tapping while wearing dentures enhanced the connectivity to the S1 (*p* < 0.05, Table 4), which tells us that somatosensory afferent pathways that contribute to perception (i.e., trigeminal sensory nuclear complex–posteromedial ventral nucleus of the Th–SI pathway).

The increased functional connections from the SI were also detected in the Bg, Cb, PMA/SMC, and DLPFC, which are thought to be primarily involved in voluntary movements. Indeed, tapping with dentures enhanced connectivity from the putamen to the M1/S1, PMA/SMC, and the cingulate cortex, but it decreased connectivity to the VL nucleus in the Th (*p* < 0.05, Figure 8 and Table 4). The brain’s circuits for voluntary action are known to consist of loops rather than linear chains; thus, this may correspond to the cerebral cortex–Bg–cerebral cortex loop (Alexander et al., 1986; Middleton and Strick, 2002).

When we defined a seed on the cerebellar cortex, functional connections were observed in the VL nucleus in the Th, M1, S1, PMA/SMC, cingulate cortex, and DLPFC (*p* < 0.05, Figure 8 and Table 4). The major influence of the Cb on movement is known through its connections in the VL nuclei of the Th, which connects directly to the motor and premotor cortex (i.e., cerebellar loop).

When defining a seed on the MI, no effective connectivity sites were detected (*p* < 0.05 uncorrected). However, when defining a seed on the PMA/SMC (i.e., BA6), there was effective connectivity with BA5, the DLPFC, and Cb (*p* < 0.05 uncorrected, Table 4). This suggests that the MI receives, as in non-human primates, two broad classes of inputs: the Bg–pre-SMA pathway–MI pathway and the parietal–premotor–MI pathway (Picard and Strick, 1996).

**FIGURE 9:**
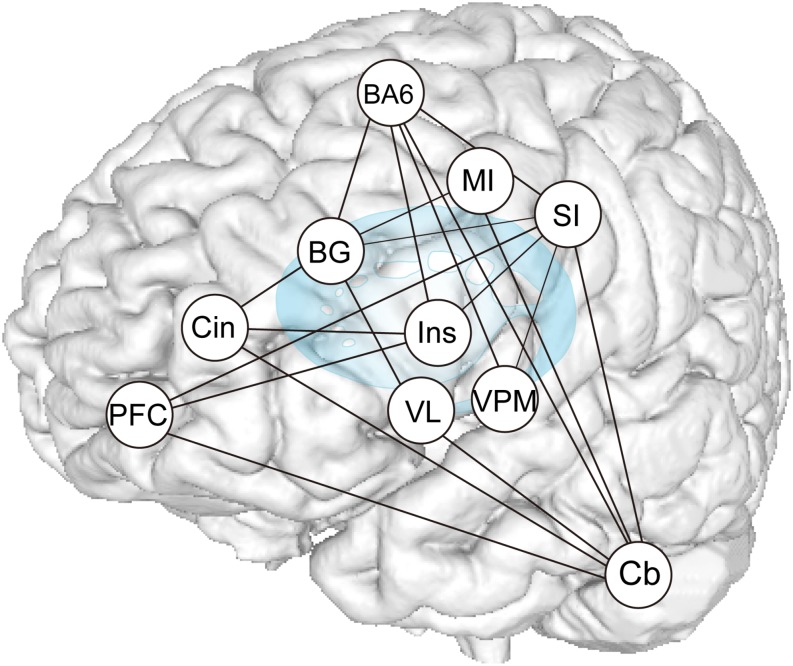
Summary of PPI analysis. Increases or decreases in functional connectivity for dentures in vs dentures out between seed regions within the distributed teeth tapping networks of the brains of elderly adult and areas related to representation, as indicated in the conjunction analysis. The sensory information used to control voluntary actions is processed in pathways that are distinct from the afferent pathways (that contribute to perception). Much oral sensory information may serve to allow for the planning and/or execution of motor acts (teeth tapping). Feedback via the cerebellar loop can be used to correct errors that arise during movement (i.e., essential smooth execution of tapping movements) and feedback via the basal ganglia loop can be used to promote sequential and rhythmical tapping.

Results of the functional connection analyses are summarized in Figure 9, which demonstrate that subcortical and cortical structures, such as the MI, SI, SMC/PMA, DLPFC, Bg, Cb, and insula cortex, probably function as hubs that form an integrated network and participate in generating and/or controlling teeth tapping.

## Discussion

Although teeth tapping appears to be a simple mechanical action, the neural machinery underlying it is surprisingly complex. Using fMRI methods, we demonstrated how elderly adults recruit various cortical and subcortical foci, including the MI, SI, prefrontal cortex, temporal association area, PFC/SMC, INS, the putamen and substantia nigra of the Bg, VPM and VL nucleus of the Th, cerebellar cortex, and amygdala, to produce tapping. When combining functional connection analyses to assess how representative foci of the cerebral cortex that are involved in the tapping task act together to execute tapping, we showed that subcortical and cortical structures, such as the MI, SI, SMC/PMA, DLPFC, Bg, Cb, and insula cortex, probably function as hubs that form an integrated network and participate in generating and/or controlling teeth tapping. All of these results support the idea that peripheral orofacial sensory inputs can notify the motor system about the current state of the body, such as its position, and movement of the teeth and jaw, and that they can allow the control of teeth tapping, for example, feedforward control of intended movements and feedback control of ongoing movements via the cerebello–cerebral loop, which can be used to correct errors that occur during movement, and feedback via the Bg–pre-SMA–MI circuit (the Bg loop), which can be used to promote smooth execution of tapping movements. This knowledge may help in explaining the increased recruitment of brain regions in the elderly compared with the young.

### How the Brain Transforms Sensory Input Into Motor Output Through a Cascade of Sensorimotor Transformations

#### How Does the Cerebral Cortex Use Sensory Information From the External World to Organize Teeth Tapping Movements?

Neural processes by which the brain controls goal-oriented behaviors, such as tasks involving arm movements and eye movements in non-human primates (for reviews, see Schwartz, 2016; Gallivan et al., 2018), have been studied well and have been reported that (1) perceptual mechanisms generate a unified sensory representation of the external world and the individual within it; (2) cognitive processes use this internal replica of the world to decide on a course of action; and (3) the selected motor plan is relayed to action systems for implementation. Teeth tapping movements may share some of the motor–visual task pathways. However, there are crucial differences between the mouth and other body parts regarding neural machinery, in terms of repetitive movements, the target, the types of sensory information being processed, and the types of joints and muscle units involved (Iida et al., 2010).

#### How Does the Brain Construct an Internal Representation of External Physical Events During Teeth Tapping?

Here, we showed changes in the activation of specific regions in the brain in the “OD *vs* OEd” condition, as well as in the “denture in *vs* out” condition. Taking into account the topographic arrangements of the body, these areas correspond to the most lateral aspect of orofacial regions (face SI). *What specific sensorimotor changes might create the brain regional activation?* “OD *vs* OEd” condition provides changes in periodontal afferents. Iida et al. (2012) reported similar cortical activation patterns during teeth tapping tasks in young adults, but they did not detect any difference in “sham teeth tapping *vs* teeth tapping task.” Therefore, the relationship between periodontal sensation and the resulting changes in tapping under experimentally anesthetized conditions remains to be examined. “Denture in (OEd) *vs* out (OE)” condition provides the following: (1) a hard vertical stop during tooth tapping, (2) changes in afferent inputs from the joints and muscles, (3) cover/mask mechanoreceptor input from periodontal ligaments and/or the alveolar ridge, and (4) changes in mechanoreceptor input from the tongue, lips, and cheeks due to material bulk. In fact, some oral sensory inputs are generated by the active movement of oral tissues, such as the contact between different surfaces in the mouth, e.g., the palate and tongue or upper and lower teeth, is rich and constant. The somatosensory experience of the mouth itself comes from the perceived object sensed by the oral tissues (for a review, see Haggard and de Boer, 2014), even though the underlying processing mechanisms of the oral sensory information remain unclear.

From studies in unanesthetized monkeys, short-train intracortical microstimulation (ICMS) was shown to evoke elemental movements, such as jaw opening and tongue protrusion, when applied to the face MI or face SI (Huang et al., 1989). It suggests that the face SI has a role in sensorimotor integration as well as the generation and control of orofacial movements.

#### How Can Sensory Feedback Be Used During Teeth Tapping Movements?

To decide on a course of action, the motor system requires information about the target object as well as the current status of the body, including its motion, posture, and location relative to the target. During hand movements, vision and proprioception provide the most prominent sources of feedback, yet they have inherent temporal delays that limit their use during very rapid corrections. Prediction can compensate for such delays and thus increase the accuracy of sensory feedback with hand movements; i.e., motor commands are copied and conveyed internally to the Cb where they are used to generate rapid predictions of the sensory consequences of motor actions (forward models; for reviews, see Scott, 2004; Scott et al., 2015). Therefore, information about the environment encoded in the sensory cortices is sent to motor systems in a feedforward fashion (for reviews, see Azim and Alstermark, 2015; Schwartz, 2016; Gallivan et al., 2018), and these sensorimotor transformations are strongly shaped by feedback from the prefrontal and association cortices to early sensory cortices. Our results support the idea that feedforward signals and sensory feedback via the cerebellar loop can be used to correct errors that arise during movement (Dean et al., 2010; Caligiore et al., 2017; Sokolov et al., 2017) and that the Cb can influence movements not only at the level of MI but also through interactions with premotor cortical areas and sensorimotor regions of the Bg (Bostan and Strick, 2018). It is important to note that the spatial location of a target can often be provided by visual input rather than somatosensation with hand movements, but within the mouth, somatosensation is the most important and vision plays a minimal role. Some oral sensory inputs are often generated by the contact between different surfaces in the mouth, such as the palate and tongue or upper and lower teeth. The somatosensory experience of the mouth itself comes from the perceived object sensed by the oral tissues (for a review, see Haggard and de Boer, 2014). Considering that the planning and execution of voluntary movements rely on sensorimotor transformations, the nervous system may have several different modes of control that use prediction and sensory feedback to different extents.

#### How Can Voluntary Action Be Executed?

A key feature of voluntary motor behaviors is that voluntary action depends on sequencing elementary movements to form a purposeful action, and the sequence of elementary movements is thought to involve parallel computations in multiple cortical areas and subcortical nuclei. To produce movements, signals from the cortex must reach motor neurons in the limbs and trunk. In humans, 30–40% of the corticospinal tract axons originate from neurons in the MI, whereas the rest of the axons have origins mainly in the SMA and PMC and in the parietal areas lying posterior to the precentral sulcus (Chouinard and Paus, 2006; Verstynen et al., 2011). Based on the finding that patients with damage to the MI are unable to independently move their fingers and can only grasp a cup clumsily, the corticospinal system is considered to not only control all aspects of body and limb movement but also have a special role in the fractionated movements necessary for skilled motor acts (for a review, see Ebbesen and Brecht, 2017). The activity of the majority of pyramidal tract neurons in the motor cortex correlates positively with movement and force (Evarts, 1968; Georgopoulus et al., 1982), suggesting a role for the motor cortex in movement initiation (Georgopoulus et al., 1982). On the other hand, damage to certain premotor cortical areas results in neurological syndromes. Both non-human primate studies and observations in humans imply a major role of the SMA in movement inhibition, and lesions to this area evoke involuntary and strange movements (for a review, see Ebbesen and Brecht, 2017). In primates and rodents, most corticobulbar and corticospinal neurons have inhibitory connections with motor neurons (Bradbury and McMahon, 2006; Isa et al., 2013).

Interestingly, long-train ICMS from the face MI and the face SI of sub-primates and non-human primates can evoke semiautomatic movements such as mastication, swallowing, and facial whisking, whereas a single transcranial magnetic stimulation of the face MI can activate one or more orofacial muscles (for a review, see Avivi-Arber and Sessle, 2018). It is considered that these semiautomatic movements are mediated by a subcortical central pattern generator (Gao et al., 2001). Animal studies have shown an intrinsic rhythmical neural pattern such that signals from cortical masticatory areas trigger the central pattern generator and execute a coordinated rhythmical pattern of jaw movements (Masuda et al., 2002; Lund and Kolta, 2006; Morquette et al., 2012). During active behavior, it is possible that the motor cortex may initiate or suppress motor programs that were initiated reflexively from subcortical circuits, such as the Bg and brainstem (Ebbesen and Brecht, 2017).

### Neural Plasticity in Oral Function

Reduced activity in elderly adults can reasonably be assumed to reflect a reduced level of functioning (Grady, 2012); however, fMRI studies have provided ample evidence for additional cortical activations in some brain areas of elderly adults (D’Esposito et al., 2003; Heuninckx et al., 2008; Grady, 2012; Samanez-Larkin and Knutson, 2015; Michely et al., 2018). During teeth tapping movements, we found an increased recruitment of brain regions in the elderly compared with young participants. An increased activity in elderly adults led to the suggestion that additional frontal activity can compensate for reduced activity elsewhere in the brain, which is beneficial to cognitive performance (for a review, see Cabeza et al., 2018). *Can age-related increases in brain activity during tapping movements be interpreted as compensation for declines in oral sensory inputs to the brain?* Alterations in oral structure and/or function (e.g., chewing and swallowing) have been shown to reshape the brain regions related to oral sensorimotor functions through a process known as neuroplasticity (Sessle et al., 2007; Avivi-Arber et al., 2011; Lin et al., 2016, 2017; Avivi-Arber and Sessle, 2018). Anatomically, connections from early sensory cortices were shown to be relayed to intermediate-level representations in the parietal lobe and then to the lateral part of the premotor cortex, which in turn projects to the MI (Rizzolatti et al., 1998). Functionally, the parietal lobe was shown to extract sensory information about the external sensory body, which is useful for the planning and guidance of movements (for reviews, see Romo et al., 2012; Azim and Alstermark, 2015; Svoboda and Li, 2018). Thus, the parietal–premotor circuit may guide teeth tapping using current oral sensory inputs.

Other interpretations of over-recruited activity in elderly adults are also possible. For example, a lack of efficiency in the use of neural resources or reduction in the selectivity of responses (which is known as dedifferentiation) can sometimes be observed with poorer performance (over-recruitment; for reviews, see Grady, 2012; Cabeza et al., 2018). Interestingly, studies employing animal models have revealed that the motor effects of motor cortical lesions greatly change over time; the acute effects of motor cortex lesions in primates and human patients are muscle weakness and slow or reduced movement. In contrast, the chronic effects are spastic hypertonia and clonus (Ebbesen and Brecht, 2017). These deficits are due to a lack of controlled movements and compromised inhibition of movement and are not due to the inability to generate movement. Indeed, numerous animal studies and classic neuropsychological studies have pointed to a major role for the frontal and prefrontal cortex in the inhibitory control of movement (for a review, see Ebbesen and Brecht, 2017). Detecting individual variations in masticatory function and/or long-term experience of mastication in the brain signatures may have a clue to understand neural mechanisms of age-related recruitment of brain regions in the elderly.

### Limitation

Although the present network analysis shows strong task-based connections between many brain regions, including MI, SI, Cb, Bg, VPM, SMC/PMA, and DLPPFC, one may point out the absence of a model. In a sense, our analysis is descriptive in nature, which is similar to the functional connectivity analysis. PPI looks very similar to comparing correlations with a seed region; however, the crucial difference is that the summary statistic reports effective connectivity, but not functional connectivity (Stephan et al., 2007; Friston, 2011). Functional connectivity analyses, e.g., principal or independent component analysis (ICA), usually entail finding the predominant pattern of correlations, or establishing that a particular correlation between two areas is significant. In contrast, effective connectivity corresponds to the intuitive notion of coupling or directed causal influence. Thus, the results of PPI may be treated in a way to identify regions whose effective connectivity with the reference region differs significantly. One can then interpret the areas that are activated by a certain task component as the element of a distributed system, though one does not still characterize how the local computations are bound together by context-dependent interactions among these areas. Another effective connectivity analysis, direct causal model (DCM), could help model comparison or optimization, although the fluctuations in the fMRI measure are typically very slow (< 1 Hz), and connectivity does not necessarily reflect monosynaptic connections (Stephan et al., 2007; Michely et al., 2018).

Contemporary theories have emphasized on how interactions among distributed neuronal populations and brain areas enable flexible cognitive operations and complex behaviors (Sporns and Betzel, 2016). Thus, analytic advancements in network science and statistics can be beneficent to quantify and represent the structural and functional connectivity of the brain and to make a feasible model for deducing its organizational properties (Bullmore and Sporns, 2009; Bassett and Sporns, 2015; Petersen and Sporns, 2015; Avena-Koenigsberger et al., 2018; see also Arce-McShane et al., 2016), even though the underlying brain circuitry that initiates and executes jaw movements remains uncertain.

## Data Availability Statement

Because of the large size of the dataset, the raw data will be made available by the authors to any researcher who makes scientifically feasible requests. The sharing of the data will occur after appropriate institutional data use agreements have been completed.

## Ethics Statement

The studies involving human participants were reviewed and approved by Medical Ethics Committee of Iwate Medical University. The patients/participants provided their written informed consent to participate in this study.

## Author Contributions

TK, HF, HK, and YS conceived and designed the study. TK and MK performed the fMRI recordings. HF, EI, KS, and YS analyzed the data. TK, HF, EI, KS, and YS generated the figure. TK, HF, and YS contributed to draft writing of the manuscript. All authors approved the manuscript for publication and agreed on all aspects of the work.

## Conflict of Interest

The authors declare that the research was conducted in the absence of any commercial or financial relationships that could be construed as a potential conflict of interest.
